# Evaluation of the Feasibility of a Two-Week Course of Aquatic Therapy and Thalassotherapy in a Mild Post-Stroke Population

**DOI:** 10.3390/ijerph17218163

**Published:** 2020-11-05

**Authors:** Carla Morer, Alfredo Michan-Doña, Antonio Alvarez-Badillo, Pilar Zuluaga, Francisco Maraver

**Affiliations:** 1 Institut Català de la Salut, EAP 8K, Centro Atención Primaria Rio de Janeiro, UTAC Muntanya, 08016 Barcelona, Spain; cmorer.bcn.ics@gencat.cat; 2 Departamento de Medicina, Instituto Investigación e Innovación Biomédica de Cádiz, Hospital Universitario de Jerez, Universidad de Cádiz, 11003 Cádiz, Spain; alfredo.michan@uca.es; 3 Department Radiology, Rehabilitation & Physiotherapy, Medicine School, Complutense University of Madrid, Plaza Ramón y Cajal s/n, 28040 Madrid, Spain; alvabadi@ucm.es; 4 Statistics and Operations Research Department, Medicine School, Complutense University of Madrid, Plaza Ramón y Cajal s/n, 28040 Madrid, Spain; pilarzul@ucm.es; 5 Professional School of Medical Hydrology, Complutense University, Plaza Ramón y Cajal s/n, 28040 Madrid, Spain

**Keywords:** aquatic therapy, thalassotherapy, stroke, balance, gait, pain, quality of life

## Abstract

Strokes are a leading cause of disability in developed countries. Patients with disabilities need rehabilitation to improve their physical functioning, mental status, and quality of life. Currently, no high-quality evidence can be found attesting the benefits of any of the interventions that are nowadays used. Water-based exercise may improve the physical conditions and quality of life of people in the post-stroke phase. The objective of this study is to test whether aquatic therapy in an enriched environment at the seaside (a thalassotherapy center) could play a role in this condition. A quasi-experimental prospective study consisting of a specific program assessed 62 patients with a mild–moderate disability pre- and post-2 weeks of intensive treatment. They followed a thalassotherapy regimen including aquatic therapy in a sea water pool at 32–34 °C for 45 min daily five times a week. The outcomes measured were the Berg Balance scale, the Timed Up and Go test, the 10-meter walking test, the 6-min walking test, the Pain Visual Analogue Scale, the WHO Well-being index, EuroQoL VAS and EuroQoL 5D. We observed a significant improvement in all outcomes measured (*p* < 0.001, except mobility EuroQoL *p* < 0.05), except in the other four dimensions of the EuroQoL 5D and 10-metre walking test (NS). Conclusion: A two-week intensive course of aquatic therapy and thalassotherapy may be beneficial in the short term by reducing pain and improving the functional status and overall well-being of post-stroke patients.

## 1. Introduction

A stroke is a neurological disease caused by the obstruction of normal blood flow due to vessel rupture or blockage, causing damage to brain tissue. Worldwide, stroke is the second leading cause of death and the third most common cause of disability, representing the first cause of severe disability of neurological origin in adults. In addition, fifty per cent of ischemic stroke survivors have a permanent disability. Although a gradual decline in mortality has been achieved in recent years, it remains the leading cause of physical disability in adults, mainly due to an ageing population [1,2,3].

Faced with this important public health problem, despite the fact that primary and secondary prevention are crucial to reducing global stroke disability, it is clear that systematic improvement in its management, including rehabilitation, can also reduce mortality and disability from this cause. On the other hand, stroke survivors routinely have motor deficiencies that cause a decrease in hemiparous leg strength and poor balance, with consequent incapacity in daily life activities and a high incidence of falls [4].

Nevertheless, since the Helsinborg Declarations (1995 and 2006), there has been no specific preponderant technique that can be recommended, although multiple methods are used effectively for the recovery of functionality and mobility after a stroke, which has led to the recommendation of a multidisciplinary approach [5].

Thalassotherapy (Greek for the Sea) consists of the medical use of marine waters, characterized by their high mineralization, high density, and chemical composition rich in chlorides, sodium, magnesium, calcium, potassium, iodine, etc., as well as of the application of sea muds called limos (pelotherapy). Further, it involves a methodical and systematic exposure to the sun and marine climatotherapy [6,7]. Aquatic therapy, according to Alonso [8], “is a therapeutic procedure in which the mechanical properties of water are used in combination with specific treatment techniques and intentions in order to facilitate the function and achievement of the proposed therapeutic objectives”, and one of the main pathologies that benefit from these techniques is the neurological processes of an adult [9], including people affected by stroke. The integration of this methodological approach might be considered according recent reviews [10,11].

After conducting a three-week pilot study [12], the objective of this study is to analyze the feasibility and the effectiveness of an intensive two-week program of thalassotherapy and aquatic therapy in a Mediterranean climate by analyzing the balance, gait, pain, quality of life, well-being and health perception of patients who have suffered a stroke.

## 2. Materials and Methods

### 2.1. Participants

Patients were recruited through press announcements, websites, patient associations and the collaboration of some provincial councils. Through a telephone interview, those who met the conditions of having had a stroke were admitted regardless of the duration of the disease or the initial level of disability, but all of them had to be clinically stable. They also had to be able to spend two weeks undertaking the treatment; these were our inclusion criteria.

The exclusion criteria were as follows: a disability classified on the Modified Rankin Scale (MRS) of 4 or more; the presence of any comorbidity that could influence his or her physical training; severe cognitive impairment and any absolute contraindication to thalassotherapy (febrile or immunodeficiency states, decompensated heart disease, severe hypertension, hyperthyroidism, immediate postoperative neoplastic processes, organic pathology, etc.) [13,14] or aquatic therapy (infectious or febrile processes, infectious diseases and contagious dermal conditions, open or healing wounds, acute phases in rheumatic processes and outbreaks in degenerative muscle diseases, severe or unstable heart or respiratory problems that may worsen with physical exertion and environmental conditions of the facility, severe renal failure, hypotension or severe hypertension or uncontrolled pressure, severe alteration of thermoregulation, etc.) [8].

**Figure 1:**
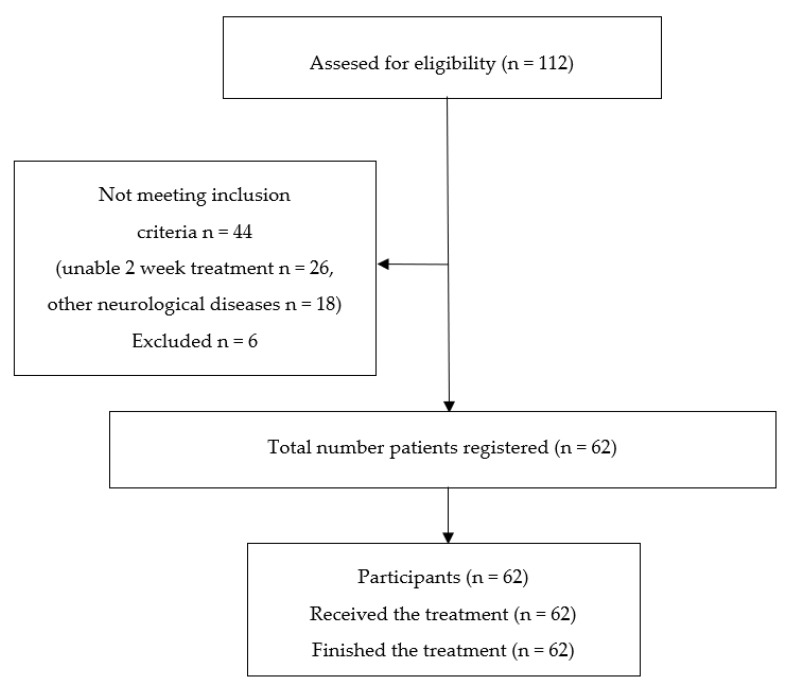
Study design flow chart.

In total, 112 patients replied, of which 26 were not able to spend the time required for treatment and another 18 had other neurological diseases (Parkinson disease, multiple sclerosis and Poliomyelitis) and did not meet the inclusion criteria. Concerning the exclusion criteria, 2 patients had heart failure NYHA 3-4, 2 were Ramkin 4, 1 had colon cancer and 1 bullous dermatitis. Finally, 62 patients were considered eligible for the start of the program. All participants included in this study gave their informed consent. The study was conducted in accordance with the regulatory standards of Good Clinical Practices and the Helsinki Declaration 2000. The selection process of the study participants is shown in Figure 1.

### 2.2. Methods

The study was designed as a prospective before and after type study in which evaluations were conducted basally and at 2 weeks after the intervention, one day before leaving. Obviously, neither the patients, the therapists nor the researchers were blind to the intervention; the expert physiotherapist who carried out the evaluations was part of the therapeutic team at the level of the management and coordination of the program, but did not perform the patients’ treatments. The study was approved by the ethics committee of the department of Physical Medicine and Rehabilitation. Medical Hydrology of Complutense University (UCM 911757).

The work lasted two weeks and was carried out at an authorized health center of Thalassotherapy of the Community of Murcia, inventoried in the Register of Regional Health Resources (RRSR) for the purposes provided for in Article 5 of Decree 309/2010, 17 December, with the number 10600001, as a Thalassotherapy Centre, being sanitarily regulated by Decree 55/1997 (on the sanitary conditions of spas, thermal baths and thalassotherapy establishments, and the application of peloids; Ministry of Health and Social Policy of the CA de Murcia) that regulates the staffing of health personnel.

### 2.3. Measurements

Descriptive measures, such as age, sex, stage of stroke (less than six months from the first stroke, between six months and 1 year, and more than 1 year) and type of stroke (ischemic or hemorrhagic) were performed. We evaluated the limitation for the Barthel Scale for Daily Life Activities as well as the Morse and Downtown Fall Risk scales for all patients in order to define the sample type. Balance was determined and evaluated by the Berg Balance Scale (BBS). Dynamic balance was measured with the Timed Up and Go Test (TUG). There are only a few standardized measures that measure mobility. In this study, we used the time necessary to cover a certain distance—short distance (time to travel 10 m) and another longer time (6 min), which also measures endurance and physical form [15,16,17].

The EQ-VAS records the self-perception of the interviewer’s health in a vertical analogue to the visual scale, in which the endpoints are labeled “best health imaginable” (100) and “worst state of health imaginable”. Moreover, patients were asked to mark the place on the VAS scale corresponding to their pain level. This scale is a valid and common tool for measuring pain intensity [18].

Another test performed was the WHO 5 Item Well-Being Index (WHO-5), which is one of the most widely used questionnaires to assess subjective psychological well-being [19], and another measure of the outcomes was health-related Quality of Life, as evaluated with EQ-5D, which exists in many languages, developed by the EuroQol group to estimate responses in 5 dimensions (mobility, activity, pain, anxiety and depression and self-care). EQ-5D is particularly useful for assessing a wide range of health conditions and treatments over time, as it provides a simple descriptive profile, but it also provides a single index value that may be used for comparisons between populations and socioeconomic data (EQ-VAS), which can be used as a quantitative measure of health outcome [20].

### 2.4. Interventions

This study was conducted in a Thalassotherapy Centre that meets the international quality standards for a mild, moderately dry and hot Mediterranean climate. The participants took part in the program in groups of up to 15 patients, always in the months of March–May or September–November, avoiding the coldest and hottest months. On day 1, the patients arrived at our center. On day 3, all the evaluations were carried out. On the 14th day, the group returned home, after the final evaluation and last medical consultation. The participants received a total of 10 sessions (over 2-weeks) from a group of interventions (5 days a week). Each session consisted of 2 elements: 10 of thalassotherapy (twenty minutes of mud or seawater bath and a one hour walk for exposition to the climate) and 10 of aquatic therapy.

All participants received a summary of the interventions performed, the improvements observed, and guidelines for continuation at home/the rehabilitation center. They were also given a discharge medical report for their GP with their evolution, overall program tolerance and possible adverse effects/medical recommendations.

#### 2.4.1. Thalassotherapy

The main “thalassohidric” factors used in our study total three:

- Seawater

Mainly, due to their mineralization, they are sodium-chloride waters, which contain virtually all elements of the periodic system, but above all chlorides, sodium, sulphates, calcium, magnesium, etc., with a residue in our environment of 35–37 g per liter, a high density (1032), and an alkaline pH of 7.5 [6,13,14]. Their characteristics, described in Table 1, were determined in our laboratory by standardized methods [21];

- Sea climate

This is characterized by its temperature, which is benign and mild, low in summer and high in winter, with very limited annual and daytime variations. The sea confers stability to the climate (as high and constant a relative humidity as the temperature, with abundant mists). In this zone, the winds alternate between sea and earth breezes, and warm and humid winds. The barometric pressure is high, close to 760 millimeters of mercury, as befits the seashore. The climate is characterized by many hours of sunshine, not only by direct solar radiation, but by the intensity of the luminous and chemical rays reflected largely by the sea and the sand itself, as well as that which is diffused by the abundant mist. All of the characteristics above described contributes to the purification of air, which is rich in oxygen and ozone, and with the presence of indications of iodine and sodium chloride [14,22]. These data are shown in Table 2.

- Sea peloid

Marine peloids, also known as lime muds (mud therapy or pelotherapy), are nothing more than the union of a solid, organic or inorganic substrate, in our case 95% phyllosilicates (total mineralogy) and 89% smectite (clay fraction mineralogy), with a liquid substrate, in our case seawater. These are characterized by having a much lower heat capacity than water, as they are bad conductors and, finally, as being able to tolerate more heat than water. The lime is applied, in essence, as thermotherapeutic agents, heated to a temperature of 45 °C, to be applied directly, either generally or partially, at 42 °C over a time of about 30 min, according to individual tolerance [23,24]. The lime’s characteristics, measured in our laboratory by standardized methods [25,26], are also shown in Table 2.

#### 2.4.2. Aquatic Therapy (Halliwick)

During the 2 weeks of the study, the 62 patients received a total of 10 sessions (5 per week), consisting of individual interventions lasting 45 min. They were performed by a physical therapist specializing in aquatic therapy linked to the center where the study was developed. The sessions were held in a seawater pool of 12 × 8 m, with a depth of 140 cm (chest depth). The water temperature was 32 °C and the ambient temperature was 24 °C. The treatment program was carried out entirely in a swimming pool specially designed to develop aquatic therapy techniques. The sessions were scheduled with a progression in difficulty. Initially, exercises were carried out for the patients to become acquainted with the water and adapt to the environment, and in the final part, stretches and relaxation techniques were performed in flotation. The practice of Halliwick Water Therapy lasted 30 min.

In detail, the Halliwick sessions were conducted by a physiotherapist who had completed a certified Halliwick Therapy Course (IATF). The sessions included time to enter and exit the pool (adapted access). The actual treatment lasted about 35 min, of which 5 min were taken to become familiar with the water and mental adaptation (Phase 1), 10 min to exercise rotational control (Phase 2), 15 min for the specific therapy based on the patient and their objectives (Phase 3), and the last 5 min were for stretching and relaxation techniques while floating with support elements [27].

### 2.5. Statistical Analysis

For each quantitative test, the number of cases (N) is described as the minimum and maximum value, as well as the mean and standard deviation (Sd) and median. All the tests used in our work are non-parametric.

We give the initial value and the value at 2 weeks, as well as two other variables that have been created, namely “salt”, which is the output value at 2 weeks when they come out, and another variable, “dif”, which is the difference between the start and end values (or end minus start for it to be positive and allow easier interpretation). If the data did not follow a normal distribution, nonparametric tests were used, specifically, the Wilcoxon test for paired samples (initial and final values). For qualitatives, frequency tables are given with input and output variables, and are compare to McNenar’s test for paired data. The comparison was considered statistically significant when *p* < 0.05. The data are presented as mean **+-** Sd. All statistical analyses were performed using SPSS (version 22.0 for Windows, SPSS Inc., Chicago, IL, USA).

## 3. Results

In total, 112 post-stroke patients were recruited for the study. Of these, 50 did not met the inclusion criteria or were excluded. Finally, 62 individuals (38 males, mean age 69, range 49–87 years) received treatment. In total, 47 patients had suffered an ischemic stroke and 15 a hemorrhagic one. In 50 cases the stroke was more than one year earlier, in 11 it was between six months and one year previous, and only 1 had it in the previous six months. These data are shown in Table 3 with the results of the Barthel, Morse Fall, Downton and Rankin scales.

Table 4 displays the scores obtained pre- and post-intervention on the Berg Balance Scale (BBS), the Timed Up and Go Test (TUG), the mobility capacity (Time to travel 10 m and 6-Minute Walk Test), the visual analogue scale of pain (VAS) and the WHO-5 well-being index. We found significant differences in all these parameters, except for the T 10-MW.

Finally, Table 5 shows the scores for pre- and post-intervention on the EuroQol VAS and on all of the sections of the EuroQol 5 Dimensions scale. Conversely, we only found significant differences in the EQ-VAS and in one of the components (mobility) of the EQ-5D. There were no significant differences in the selfcare, visual activities, pain/discomfort or anxiety/depression areas.

## 4. Discussion

This study investigated the effectiveness of a program of aquatic therapy and thalassotherapy in a Mediterranean climate on the balance, mobility, pain, quality of life, well-being and health perception of stroke patients.

Gait training is the most common rehabilitative intervention performed by the post-ictus patient. There are systematic reviews that have considered the benefit of fitness training in the gym by combining or comparing rolling tape training or body weight support tape [28,29]. According to some recent research, the post stroke patients consume less energy in chest-depth water, which may allow them to perform the training for a prolonged duration [30].

Even more revealing is the research on the impact of vertical immersion in water for healthy subjects, showing positive effects on cerebral circulatory flows, cortical activation, executive functions, and neurotrophin production [31]. Some studies also reveal the effects of exercise in water on cerebral arterial circulation, as increases in carotid diameter and blood flow rate have been reported at about 7% which lasts during the exercise in water, compared to dry exercise at the same intensity [32]. Moreover, the synthesis of endothelial nitric oxide increases during exercise in water, allowing a vasodilator response of the smooth vascular musculature by lowering blood pressure [33]. In this context, the Sato et al. group published some studies on immersion in water that suggest that it can improve the effectiveness of physical therapy in stroke rehabilitation. It activates the affected areas of the cerebral cortex, thus improving signal processing and cortical learning (cortical processing of somato-sensory inputs), as well as having the added benefits of water flow stimulation (and not just with immersion) in intra-cortical circuits and changes in cortico-spinal excitability, increasing the activation of the motor cortex for the planning and execution of voluntary movements [34,35]. Moreover, treatments carried out in an aquatic environment have shown their benefits in numerous neurological processes [36,37,38].

In this context, aquatic therapy has been found beneficial for many aspects of the functional limitations observed following a stroke, including paralysis and weakness, balance dysfunction, and gait disturbances [10,39]. In contrast, to our knowledge, there are only a few studies on the effect of thalassotherapy in these patients [12,40,41].

Our findings show that the use of both aquatic therapy and thalassotherapy in the treatment of stroke is beneficial for improving the functionality (balance and mobility), pain, and some aspects of the quality of life. We are going to discuss these in four sections, as follows: 1. Balance, 2. Mobility, 3. Pain and 4. Quality of life.

### 4.1. Balance

It is a well-known fact that an aquatic environment produces buoyancy, reducing the impact of gravity, while also adding the effects of viscosity to the effort of movement, allowing for an increase in the joint range and the muscle strength in the lower extremities, both of which have rehabilitative implications.

Water exercise, in fact, makes it easier for stroke patients, who usually have difficulty managing their entire weight on their lower limbs, to walk. To this end, water buoyancy allows post-ictus patients to move with less effort and planes that would be impossible while unassisted on land. In this way, a training program based on repetition and intensity can cause substantial changes in the patient’s sense of balance. In addition, an aquatic environment provides a safe environment for the patient by minimizing the risks and consequences of a possible fall [42,43].

Accordingly, it is justified to state that both static and dynamic balance is improved, as assessed by the BBS, (*p*-0.000) and by the TUG, (*p*-0.004), respectively.

### 4.2. Mobility

Aquatic therapy is probably the simplest method to promote mobility, so it seems plausible that performing aquatic therapy and thalassotherapy improve the characteristics of gait in post-stroke patients, especially in those with greater difficulties.

Based on the fundamentals described for balance, we tested this possibility, and we did not find a significant result over a short distance (T 10-MW, *p*-0.115), but we did find a significant one for a longer running time (6-MWT, *p*-0.000). Previously, Chu et al. [44] showed that an eight-week course of aquatic therapy was more effective versus a control group in increasing gait speed, muscle strength in the lower lims, and the cardiovascular physical condition in post-stroke patients. However, a Cochrane systematic review does not confirm or refute that such exercises in water could help decrease post-ictus disability. We should point out that this was done in 2011, and the data come from studies with small samples [36]. On the other hand, Furnari et al. [45] demonstrated that hydrokinesitherapy, combined with dry physical exercise, significantly improved the balance and the pattern of gait in post-stroke (subacute) patients, and, furthermore, in a 2018 randomized study in a cohort of 20 subacute stroke patients, following a 30-min, 6-week, five-times per week comparison assessing land-based aerobic exercise with aquatic treadmill exercise, investigators found that the aquatic group showed greater gains in the 6-min walk test, a peak O2 uptake, and increases in the endurance exercise measure [46].

### 4.3. Pain

One of the goals of stroke treatment is also to mitigate pain, as this not only decreases the patient’s quality of life, but also prevents the execution of rehabilitation activities, and conditions the results of the patient. It may manifest as allodynia (an abnormal situation in which a non-harmful stimulus is perceived as painful) and/or hyperalgesia, and/or hyperpathy (abnormally intense painful response to harmful stimuli). Pressure and temperature sensitivity are often altered too. Patients often show autonomic alterations in the painful region with vasomotor instability. No therapy strategy is effective for all patients, so it is not uncommon to combine different treatment modalities (medication, physiotherapy, massage, exercise, etc.). Despite the importance of mitigating pain for the development of a stroke rehabilitation program, previous published studies on aquatic therapy have not analyzed this outcome [42]. This pain not only limits the quality of life, but also the intensity of rehabilitation and its results.

Although the EQ-5D pain/discomfort dimension did not improve significantly, the Visual Analogue Scale (VAS) manifested a marked improvement (*p* < 0.001). In this way, the patients experienced improvements in overall pain, probably due to multiple factors. In our opinion, these data are especially relevant, since, with the background of health resort medicine in this field, the thalassotherapy interventions were aimed at this purpose (to improve pain and to be able to continue the program at the desired intensity). These results are also consistent with recent studies performed on other modalities of aquatic therapy [47,48].

### 4.4. Quality of Life

We used the validated versions of the EQ-VAS and EQ-5D questionnaires. Both of these are assessment tools for evaluating quality of life, which are both easy to administer and clear. Their psychometric properties had been widely studied, with proven reliability, validity and sensitivity for the general population, and for patients with different disorders. Specifically, the EuroQoL multidimensional questionnaire evaluates the positive and negative aspects of health. It consists of 25 items comprising, five domains, as follows: mobility, self-care, usual activities, pain/discomfort and anxiety/depression.

Although a previous work has described the positive effects of physical exercise performed in water improving the mood and quality of life of people with acquired brain injuries [48], our data did not show similar results. Some circumstances may explain this disparity, for example, their treatment program had a longer duration (12 weeks), combined dry land with aquatic physiotherapy (Ai Chi), and was applied in a younger population (mean age 61.4 versus 69 years). In a similar way, Temperoni et al. showed in a controlled randomized trial that a sequential preparatory approach added to a water-based exercise program, in line with the suggestions of the Hydrotherapy Association of Chartered Physiotherapists Guidance on Good Practice in Hydrotherapy but with a different method from the Halliwick one, the effectiveness of aquatic therapy on stroke patients’ quality of life [49]. In this sense, other investigations have not found positive results. An 18-month program of aquatic therapy among a group of stroke patients failed to find an advantage for the control of depression and anxiety [50]. In this sense, we used the WHO-5 Scale and we found significant improvements. Well-being is crucial for stroke rehabilitation, and thalassotherapy and health resort medicine may play a role on this [7,51].

This study has several limitations. First, the lack of a control group. A larger randomized controlled clinical trial is needed to validate the benefits reported in our study. In addition, the evaluation of the scale was not blind, as this limitation may include a bias in the study. Finally, our treatment requires the transportation of the patient from his/her home to the therapeutic center with a Mediterranean climate, which makes it impossible to provide a subsequent follow-up evaluation. The absence of this follow-up is another limitation, which should be considered in further related studies.

## 5. Conclusions

In conclusion, the results of this study indicate that an aquatic therapy and thalassotherapy program undertaken in a Mediterranean climate may be feasible and may have positive effects on the balance, mobility and pain, and on certain elements affecting the quality of life, well-being and health perception, of people who have suffered a stroke with mild disability. Therefore, health professionals working in the field of neuro-rehabilitation should at least consider this treatment option. Additional large, multicenter trials should be conducted to investigate how aquatic therapy and thalassotherapy can be incorporated into the current rehabilitation programs.

## Figures and Tables

**Table 1:** Thalassotherapy factor: seawater.

Seawater	Jan	April	July	Oct	Mean
Conductivity to 25 °C (μS·cm^−^^1^)	67,300	65,600	66,700	67,280	66,720
pH	7.4	7.5	7.5	7.6	7.5
Li^+^ (mg/L)	0.09	0.08	0.09	0.09	0.09
Na^+^ (mg/L)	11,343	11,570	11,643	11,426	11,496
K^+^ (mg/L)	472	459	470	444	462
Mg^2+^ (mg/L)	1405	1417	1431	1331	1396
Ca^2+^ (mg/L)	562	537	511	548	540
F^−^ (mg/L)	1.5	1.5	1.4	1.5	1.5
Cl^−^ (mg/L)	19,541	19,103	19,586	20,482	19,678
Br^−^ (mg/L)	6.7	6.6	6.0	6.1	6.4
HCO_3_^−^ (mg/L)	134	140	134	140	137
NO_3_^−^ (mg/L)	10	10	10	10	10
SO_4_^2^^−^ (mg/L)	2833	2713	2837	2741	2782

**Table 2:** Thalassotherapy factors: sea climate and sea peloid.

Sea climate	Jan	Feb	Mar	April	May	June	July	Aug	Sept	Oct	Nov	Dec	Mean
Temperature													
Average temp. (°C)	11.1	12	14.2	16.2	19.3	23.1	25.8	26.2	23.7	19.5	15.4	12.4	18.2
Min. temperature (°C)	6	6.7	8.8	11	13.9	17.5	19.9	20.4	18.1	14.2	10.2	7.6	12.9
Max temperature (°C)	16.2	17.3	19.7	21.5	24.8	28.8	31.7	32	29.4	24.8	20.6	17.3	23.7
Pluviometry													
Precipitation (mm)	24	19	23	27	25	13	4	7	29	55	38	35	24.9
**Sea peloid**													
Centesimal composition				Instrumental texture						Thermal properties			
Water (%)	59.9			Hardness (g)			45			Specific heat (C_p_)			2.9
Solids (%)	40.1			Adhesiveness (g s)			548			Relaxation time t_r_ (s)			498
Ash (%)	35.8			Cohesiveness			0.96			Heat amount Q (J)			16,644
Ash/Solids	0.88			Adhesiveness/Hardness			12.10			Heat flow Φ (J/s)			33.4

**Table 3:** Description of the participants.

Characteristics	Participants
Sex	Females 24 (38.7)
	Males 38 (61.3)
Age (years)	Min. 49
	Max. 87
	Mean 69
Stroke type	Ischemic 47 (75.8)
	Hemorrhagic 15 (24.2)
Stage	<6 months: 1 (1.6)
	6 months–12 months: 11 (17.7)
	>1 year: 50 (80.7)
Barthel Scale	Min. 15
	Max. 100
	Mean 70.3
Morse Fall Scale	Min. 20
	Max. 90
	Mean 67.7
Downton Scale	Min. 3
	Max. 7
	Mean 4.24
Rankin Scale	Min. 1
	Max. 3
	Mean 1.92

**Table 4:** Pre- and post-intervention scores.

	Min.	Max.	Mean	Sd	Median	*p*-Value
BBS						
Pre	0.0	56.0	25.8	19.2	28.5	
Post	0.0	56.0	31.3	19.6	36.0	
Mean diff.	−2.0	23.0	5.5	5.2	5.0	*p* < 0.001 *
TUG						
Pre	6.0	240.0	49.2	45.8	43.0	
Post	6.0	144.0	39.7	31.3	36.0	
Mean diff.	−5.0	96.0	9.4	18.9	3.0	*p* < 0.001 *
T 10-MW						
Pre	6.9	240.0	43.0	47.8	26.5	
Post	5.9	165.0	38.1	36.4	21,5	
Mean diff.	−71.0	75.0	4.8	25.2	1.0	*p* = 0.115
6-MWT						
Pre	0.0	630.0	153.4	153.9	113.0	
Post	0.0	683.0	180.0	170.6	135.0	
Mean diff.	−80.0	139.0	27.3	39.0	15.0	*p* < 0.001 *
VAS						
Pre	0.0	10.0	3.0	3.4	0.0	
Post	0.0	9.0	1.7	2.4	0.0	
Mean diff.	−3.0	8.0	1.2	2.1	0.0	*p* < 0.001 *
WHO−5						
Pre	20.0	100.0	62.8	21.9	70.0	
Post	28.0	100.0	78.4	16.6	80.0	
Mean diff.	−4.0	48.0	15.5	15.0	12.0	*p* < 0.001 *

BBS = Berg Balance Scale, TUG = Timed Up and Go, T 10-MW = Time to Travel 10 m, 6-MWT = 6-Minute Walk Test, VAS = Visual Analogue Scale, WHO-5 = WHO 5 Item Well-Being Index, Significance. level: * = *p* <0.001.

**Table 5:** EuroQol Visual Analogue Scale and 5 Dimensions, pre- and post-intervention scores, showing the scores for EQ-VAS and frequency tables for EQ-5D.

	Min.	Max.		Mean	Sd	Median	*p*-Value
EQ-VAS							
Pre	0.0	91.0		57.1	19.2	60.0	
Post	20.0	95.0		66.1	15.4	65.0	
Mean diff.	–25.0	46.0		8.9	12.2	9.0	*p* < 0.001 *
	Post	0.0	1.0	2.0	3.0	Total	*p*-Value
EQ-5D Mobility							
Pre	0.0	0	0	0	0	0	
	1.0	0	14	0	0	14	
	2.0	0	6	38	0	44	
	3.0	0	0	1	3	4	
Total		0	20	39	3	62	*p* = 0.030 **
EQ-5D Self-care							
Pre	0.0	1	0	0	0	1	
	1.0	0	30	1	0	31	
	2.0	0	2	18	3	23	
	3.0	0	0	3	4	7	
Total		1	32	22	7	62	*p* = 0.846
EQ-5D Usual activities							
Pre	0.0	1	0	0	0	1	
	1.0	0	15	4	1	20	
	2.0	0	8	19	1	28	
	3.0	0	3	3	7	13	
Total		1	26	26	9	62	*p* = 0.343
EQ-5D Pain/discomfort							
Pre	0.0	1	1	0	0	2	
	1.0	0	21	2	0	23	
	2.0	0	2	26	0	28	
	3.0	0	0	4	5	9	
Total		1	24	32	5	62	*p* = 0.172
EQ-5D Anxiety/depression							
Pre	0.0	1	0	0	0	1	
	1.0	1	30	3	0	34	
	2.0	0	6	17	0	23	
	3.0	0	0	1	3	4	
Total		2	36	21	3	62	*p* = 0.392

EQ-VAS = EuroQol Visual Analogue Scale, EQ-5D = EuroQol 5 Dimensions (0 = no problems; 1= slight problems; 2 = moderate problems and 3 = severe problems), Significance level: * = *p* < 0.001, ** = *p* < 0.05.
